# International Clinics

**Published:** 1891-06

**Authors:** 


### Book Reviews.

INTERNATIONAL CLINICS. J. B. Lippincott Company, New York.

We have before us a work which bids fair to prove of inestimable value to physicians—especially the busy ones. As set forth on its title page, it is, "A quarterly of clinical lectures on Medical Surgery, Gyneacology, Pediatrics, Neurology, Dermatology, Laryncology, Opthalmology, and Otology, by Professors and Lecturers in the leading medical colleges of the United States, Great Britain and Canada. It is edited by four excellent men—headed by John M. Keating, of Philadelphia. It proposes to cull from all the various clinical lectures the best in every branch, and to furnish reading matter which will be of the most practical value to the physician. It proposes to make the series a complete post-graduate medical course. It will supply a long felt want, for we know that there are a great many physicians whose circumstances are such as to render it almost impossible for them to pay an annual visit to some of the large cities where they may obtain the advantages of a post-graduate instruction. These clinics on paper give them in a concise form, the latest medical knowledge from the most advanced thinkers. We are much pleased with its make-up, and predict for it a prosperous future. R.

